# Structure and Synaptic Function of Metal Binding to the Amyloid Precursor Protein and its Proteolytic Fragments

**DOI:** 10.3389/fnmol.2017.00021

**Published:** 2017-01-31

**Authors:** Klemens Wild, Alexander August, Claus U. Pietrzik, Stefan Kins

**Affiliations:** ^1^Heidelberg University Biochemistry Center (BZH), University of HeidelbergHeidelberg, Germany; ^2^Division of Human Biology and Human Genetics, Technical University of KaiserslauternKaiserslautern, Germany; ^3^Institute for Pathobiochemistry, University Medical Center of the Johannes Gutenberg-University MainzMainz, Germany

**Keywords:** amyloid precursor protein (APP), zinc, copper, synaptic transmission, Alzheimer’s disease

## Abstract

Alzheimer’s disease (AD) is ultimately linked to the amyloid precursor protein (APP). However, current research reveals an important synaptic function of APP and APP-like proteins (APLP1 and 2). In this context various neurotrophic and neuroprotective functions have been reported for the APP proteolytic fragments sAPPα, sAPPβ and the monomeric amyloid-beta peptide (Aβ). APP is a metalloprotein and binds copper and zinc ions. Synaptic activity correlates with a release of these ions into the synaptic cleft and dysregulation of their homeostasis is linked to different neurodegenerative diseases. Metal binding to APP or its fragments affects its structure and its proteolytic cleavage and therefore its physiological function at the synapse. Here, we summarize the current data supporting this hypothesis and provide a model of how these different mechanisms might be intertwined with each other.

## Introduction

Alzheimer’s disease (AD) is a fatal neurodegenerative disorder and a severe burden of our aging societies (Schaller et al., 2015). One pathological hallmark of AD is the formation of senile plaques deposited in the brain concomitant with a massive decline of neuronal mass and therewith of memory and cognitive abilities (Selkoe and Hardy, 2016). On the molecular level, the amyloid precursor protein (APP) is fundamental to the pathology as the plaques predominantly consist of its proteolytic breakdown product, which is the neurotoxic amyloid-beta peptide (Aβ; Selkoe, 2011; Haass et al., 2012). APP and the paralogous APP-like proteins (APLP1 and 2) are expressed in various tissues (APLP1 only in the brain) and in alternative splice forms (Walsh et al., 2007; Müller and Zheng, 2012) and are concentrated in the synapses of neurons. They are single-span type I transmembrane proteins with a large extracellular domain (ectodomain) and a short cytoplasmic tail APP intracellular domain (AICD; Coburger et al., 2014). APP is a prime example for ectodomain shedding by α-, β-, or γ-secretases and for regulated intramembrane proteolysis (RIP) by the γ-secretase complex (Thinakaran and Koo, 2008; Haass et al., 2012). In the first step, the majority of the extracellular domain is shed off at distinct sites within the juxtamembraneous domain by different proteases, involving ADAM10 and BACE. The resulting extracellular cleavage products are released in form of soluble fragments, designated according to the cleavage site as sAPPα and sAPPβ, respectively (Brunholz et al., 2012). More recently, an additional more N-terminally located η–cleavage site was described, possibly mediated by cleavage of MT5-MMP (Willem et al., 2015). This causes an even more complex picture of APP processing, including besides sAPPη different extracellularly released fragments, such as Aη–α or Aη–β peptides. In fact, accumulating evidence suggests that the APP processing is likely even more complex, as additional cleavage sites within the Aβ domain by e.g., Meprin β have been described (Bien et al., 2012). However, the different residual carboxyl-terminal fragments (CTFs) are subsequently cleaved by the γ-secretase complex, which causes in case of preceded BACE1 shedding release of various Aβ peptides (Aβ1–36 to Aβ1–43) and the AICD (Steiner et al., 2008). The regulation of cleavage is a key event for both physiological and pathological processes, whereby the pathophysiological relevance of all the different peptides is yet not well understood, and depends on the localization of APP, on post-translational modifications, and on its oligomerization state (Kienlen-Campard et al., 2008; Eggert et al., 2009; Haass et al., 2012; Muresan and Ladescu Muresan, 2015; Winkler et al., 2015). APP is in equilibrium between monomeric and dimeric species (Soba et al., 2005; Isbert et al., 2012) and underlies a rapid turnover from the cell surface into endosomal compartments, the presumed major place of Aβ generation (Thinakaran and Koo, 2008; Haass et al., 2012).

APP localization, oligomerization state and processing are influenced by direct binding to copper and zinc ions (Acevedo et al., 2011; Baumkötter et al., 2014; Mayer et al., 2014) and dysregulation of copper and zinc homeostasis are an apparent feature of neurodegenerative diseases, including AD. However, as APP binds copper and zinc with affinities in the nano and micro molar range, respectively, and as local concentrations of copper and zinc can vary quiet a lot, it appears reasonable that metal binding occurs only under certain pathophysiological conditions. Here, we summarize recent advances in the molecular details of copper and zinc binding to APP and their potential impact on APP processing and their potential role in the pathological as well as in the physiological context.

## APP and Its Fragments as Metalloproteins

**Figure 1:**
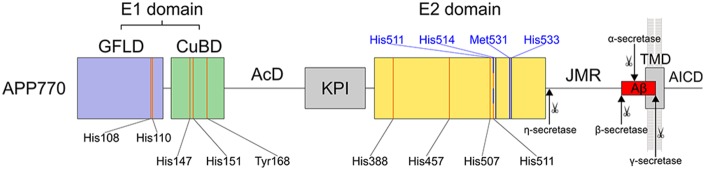
**Schematic overview of amyloid precursor protein (APP770).** The domain architecture is given with residues involved in metal binding and cleavage sites of various secretases. The E1 domain contains two copper binding sites (orange lines) with clustered residues (four histidines and one tyrosine), one in the growth factor like domain (GFLD, blue) and one in the copper binding domain (CuBD, green). The E1 domain is followed by an acidic domain (AcD) and a Kunitz protease inhibitor (KPI) domain (gray). The E2 domain (yellow) binds copper via four histidine residues (orange lines). Zinc binding involves three residues of the copper binding site (His457, His507 and His511, while His388 is replaced by a water molecule). The residues corresponding to the recently identified zinc binding site in APLP1 are highlighted in blue. Three of four histidines are conserved while His450 is replaced by Met531 in APP770. The juxtamembrane region (JMR) and transmembrane domain (TMD, gray) harbor the Aβ region (red). After β-secretase cleavage at Met671 and further γ-secretase cleavage at various sites within the TMD, Aβ is liberated into the extracellular space, whereas the APP intracellular domain (AICD) is released into the cytosol. Cleavage by α-secretases occurs within the Aβ region at Lys687. Additionally, η-secretase cleaves the ectodomain at position Asn579.

APP is a multi-domain membrane protein with a single transmembrane domain (TMD) and several unstructured regions (APP numbering in the following corresponds to APP770, UniProtKB: P05067; Figure 1). The large extracellular ectodomain (residues 18–699) is divided into the N-terminal growth factor-like domain (GFLD, 18–123), the copper-binding domain (CuBD, 124–189), an unstructured acidic domain (AcD, 190–289), a Kunitz-type protease inhibitor (KPI) domain with an Ox2 region (290–364, not present in neuronal APP695 splice form), the E2 domain (365–575), and the juxtamembrane region (JMR, 576–699). GFLD and CuBD form a structural unit termed E1 domain. The JMR harbors the sites for secretase cleavage during the process of ectodomain shedding.

**Figure 2:**
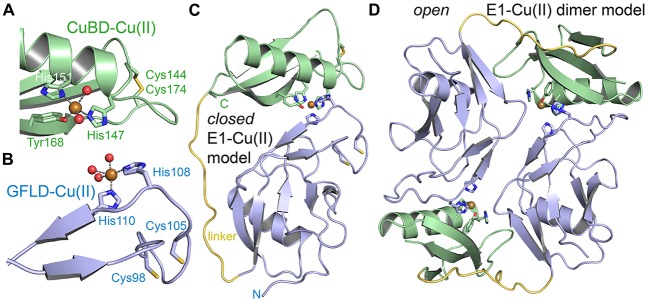
**Metal binding to the E1 domain. (A)** The CuBD of human APP bound to copper (II) (PDB code: 2fk1). Copper is coordinated by three protein ligands and two water molecules. The shown disulfide bridge is partially reduced. **(B)** The GFLD of human APP bound to copper (II) (PDB code: 4jfn). Copper is coordinated in the same geometry as in the CuBD. Two ligand sites are occupied by protein and three sites by a crystal contact (aspartate) replacing three water molecules. The inferred hydrated state in solution is shown. The disulfide bridge adjacent to the copper binding site is reduced. **(C)** A putative “closed” E1 domain by the intramolecular combination of the two ligand bindings sites of GFLD and CuBD and modeling of a flexible linker (yellow). **(D)** The respective “open” E1 domain by intermolecular combination of the binding sites. The monomeric E1 domain corresponds to PDB code 4pwq.

Metal binding is well documented to APP and to Aβ peptides (Hesse et al., 1994; Talmard et al., 2007; Kong et al., 2008; Dahms et al., 2012; Baumkötter et al., 2014). Copper binding to APP has been first structurally characterized for the CuBD (Barnham et al., 2003; Kong et al., 2007). Copper (II) binds with high affinity (*K*_D_ of 10 nM; note: binding constants to copper and zinc ions strongly depend on the applied method and conditions and values have to be evaluated critically) in a slightly distorted square pyramidal geometry (a type 2 non-blue site) to three protein ligands (His147, His151 and Tyr168) and two water molecules (Figure 2A; Kong et al., 2008). The distorted type 2 geometry is consistent with an observed redox-activity of APP, and CuBD was found to also bind copper (I) (with one water ligand lost) although no conformational change within the protein could be detected. Interestingly, although not interpreted in the original articles, a nearby disulfide bridge (Cys144-Cys174) is partly reduced in the high-resolution structures. The mechanism of how copper-binding to the CuBD could decrease Aβ production as described in CHO cells (Borchardt et al., 1999) and various mouse studies (Bayer et al., 2003) however remained enigmatic. Specific binding of zinc to two C-terminal cysteines (Cys186 and Cys187) within the CuBD has also been reported (Bush et al., 1993). However, data had been acquired with tryptic and synthetic peptides and in available structures the two cysteines are not available for zinc coordination. Crystallographic analyses of the CuBD with bound zinc would help to clarify this point and would build the basis for follow-up studies, addressing the impact of zinc binding on APP pathophysiology.

Recently, we reported specific copper binding to the N-terminal GFLD (Figure 2B; Baumkötter et al., 2014). Here, copper (II) was found to adopt the same ligand geometry with however two protein ligands (His108 and His110) in a flexible hairpin loop also involved in Heparin binding and putatively three water ligands (mimicked in the crystal by an aspartate of a symmetry contact). Binding affinity as determined by isothermal titration calorimetry (ITC) is in the low nanomolar range (*K*_D_ of 28 nM) almost as high as to CuBD. Copper-binding to the GFLD again correlates with the reduction of a neighboring disulfide bridge (Cys98-Cys105) pointing to redox-activity also of this site. Furthermore, the match of geometry and functionality is suggestive for a complementation of the two copper-binding sites by either a conformational change within the E1 domain into a “closed” conformation (Figure 2C) or by dimerization of two “open” E1 domains *in trans* (Figure 2D). In line, conformational flexibility within the E1 domain has been validated recently (Hoefgen et al., 2015). In contrast to the CuBD and E2 domains, no binding to the GFLD could be detected for zinc ions (Baumkötter et al., 2014).

**Figure 3:**
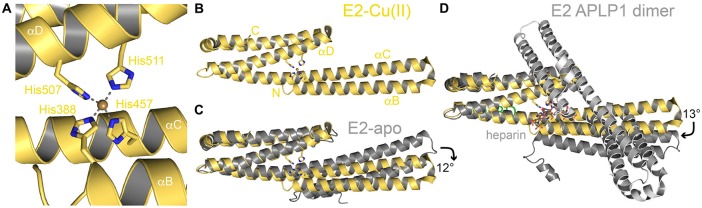
**Metal binding to the E2 domain. (A)** The copper (II) binding site in the center of the coiled-coil like human E2 domain (PDB code: 3UMK). The four histidine ligands are spread on three different helices. **(B)** Structure of the complete human E2 domain bound to copper (II). **(C)** Superposition of E2-Cu(II) with the respective apo form (PDB code: 3NYJ) highlighting the metal induced conformational change. **(D)** Superposition of E2-Cu(II) with a dimeric APLP1 E2 domain in complex with a heparin hexa-saccharide (PDB code: 3qmk). Heparin binds in a 1:2 molar ratio and occupies the copper (II) binding site.

The E2 domain (Dahms et al., 2012), which like E1 binds strongly to copper (*K*_D_ of 13 nM) and with low affinity to zinc (*K*_D_ of 3.9 μM; with some uncertainties of the ITC measurements) harbors two or three different metal binding sites, here designated as M1–3 (also see Table 1). Both metals bind to the same evolutionary conserved site (denoted as M1) within the APP family and due to the more than 100 times lower affinity, zinc (II) cannot compete for copper (II) binding *in vitro*. In X-ray structures of the metal-bound E2 domain the M1 site is found central to the coiled-coil like fold and consists of four histidines (His388, His457, His507 and His511 for APP770 numbering) spread on three helices (αB, αC and αD; Figures 3A,B; Dahms et al., 2012). The ligand coordination has been described as tetrahedrally-distorted square planar geometry (although a fifth ligand is missing here compared to the E1 copper binding sites). Notably, in contrast to the GFLD and CuBD, no redox-activity has been observed for the M1 site. The central location of the M1 site enables a metal dependent conformational switch within the E2 domain with a 12° bending of the helical rod (Figure 3C) and bending correlates with an increased rigidity and thermostability of the E2 domain.

**Table 1:** **Metal binding properties of amyloid precursor protein (APP)/APP-like proteins (APLPs)**.

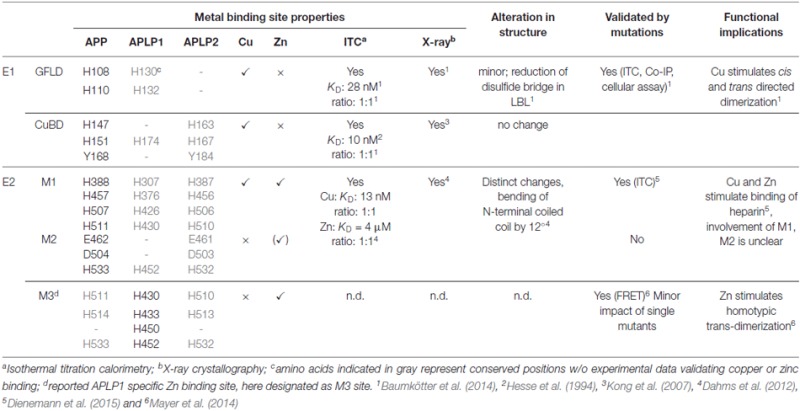

Copper binding to the E2 domain was reported to stimulate heparin binding, which might be of physiological importance by modulating APP binding to the extracellular matrix during brain development (Dienemann et al., 2015). However, in an X-ray structure of dimeric human APLP1 E2 domain bound to a heparin-hexasaccharide, the M1 site is destroyed and the four histidines are involved in carbohydrate binding instead (Lee et al., 2011). Here, the E2 domain is bent in an orthogonal direction by 13° due to dimer formation that allows the accommodation of the ligand in the dimer interface in a 2:1 protein to ligand ratio (Figure 3D).

Binding to the M2 site within the E2 domain has been analyzed by competitive cadmium displacement studies similar to analysis of M1 (Dahms et al., 2012). Thereby, cadmium was only partially replaced by zinc, but not by copper. However, no further functional studies validated its functional or structural relevance yet.

Two other more recent studies suggested a zinc binding site in the APLP1 E2 domain (between M1 and M2 of APP E2: APLP1 residues His430, His433, His450 and His452) to regulate homo- and hetero-dimerization with APP and APLP2 (Mayer et al., 2014, 2016). Notably, only three of the four histidine residues involved in binding are conserved in APP and APLP2 (Table 1). However, as mutations of single histidine residues only had a minor impact on APLP1 cell adhesion features, it appears reasonable to postulate that binding of zinc to the M3 site might affect APP and APLP2 function in a similar manner. Moreover, specific dimerization implies complementation of metal coordination in *trans*, which would require a different dimerization as observed in the X-ray structure with heparin. Such zinc mediated dimerization was observed in a crystal contact of another recent APLP1 E2 structure bound to a heparin dodecasaccharide (Dahms et al., 2015), which however, was mediated again by other surface exposed histidines. Therefore, the structural and physiological consequences of copper and zinc binding to the E2 domain appear still highly controversial and need further investigations.

Since copper and zinc binding to APP is predominantly mediated by histidine and due to pKa values of this amino acid, the interaction of APP with copper and zinc is impossible under acidic conditions. APP is trafficked through the secretory pathway to the cell surface up to endosomes and lysosomes. Thereby it passes cell compartments with different pH values. The pH of the ER is near neutral, while the downstream compartments (*cis*-and *trans*-Golgi, secretory vesicles) become progressively more acidic down to pH 6 in the *trans*-Golgi and <5 in lysosomes (Casey et al., 2010). Thus, it is likely that APP binding to copper and zinc varies between different subcellular compartments, possibly altering its structure and binding properties. In line with this, it was reported that APP can adopt different conformations depending on pH (Hoefgen et al., 2015). Interestingly, the altered structure at acidic pH is stabilized by hydrogen bonds involving His147 and Tyr168 in the CuBD that mediate copper binding under neutral pH conditions.

**Figure 4:**
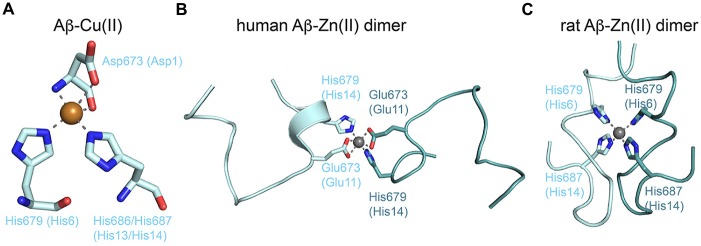
**Metal binding to the Aβ peptide. (A)** The proposed copper (II) coordination by the N-terminus of Aβ and two histidines. The model corresponds to copper (II) coordination found in the crystal structure of GFLD-Cu(II) with the aspartate originating from a crystal contact (PDB code: 4jfn). **(B)** NMR structure of dimeric human Aβ (1–16) bound to zinc (II) (PDB code: 2mgt). The zinc ion mediates dimerization via coordination of equivalent residues. **(C)** NMR structure of dimeric rat Aβ (1–16) bound to zinc (II) (PDB code: 2li9). Dimerization via the zinc ion is different and highlights the conformational flexibility of Aβ peptides.

Most attention has been given to copper and zinc binding to Aβ peptides due to the direct influence on pathological processes and metal accumulation (millimolar range) in the amyloid plaques (for review see Tõugu et al., 2011; Tiiman et al., 2013). From a structural viewpoint, the peptide-ligand complexes are difficult to tackle, as both metals rapidly precipitate Aβ and induce a multitude of Aβ conformations and oligomeric assemblies. Furthermore, Aβ conformations strongly depend on the conditions (e.g., pH and solvent) used for structure determination. The role of metal ions in the self-assembly of Aβ has been reviewed in detail recently (Faller et al., 2013). In consensus, copper and zinc bind to the N-terminus of Aβ(1–16; numbering for Aβ-peptide only) in a 1:1 ratio mostly involving the aspartate and alanine at the very N-terminus (Asp672 or Asp1 in Aβ nomenclature, and Ala673), glutamate 11, and various histidines (His6, His13 and His14). Furthermore, redox-active copper is able to induce reactive oxygen species (ROS) that lead e.g., to aggregation-prone cross-linked Aβ dimers by oxidization of Tyr10 (Smith et al., 2007). Respective residues 1–10 are missing in APLP1 and also APLP2 has a two-residue deletion including His6. Affinities for copper vary significantly between attomolar and micromolar values, although careful analyses suggest values in the range of 30–60 nM (Tõugu et al., 2011; Tiiman et al., 2013). No X-ray or NMR structure has been reported for an Aβ-copper complex. At a physiological pH of 7.4 a square-pyramidal coordination is assumed including Asp1 and His6 and His13 or His14 (Faller et al., 2013). Interestingly, this coordination is exactly as found for the X-ray structure of the GFLD-Cu(II) complex, which might therefore be regarded as first atomic model of an Aβ-Cu(II) complex (Figure 4A). Ligands might originate from the same or different Aβ peptides, which reflects the ability of copper (II) to bind to all forms of monomeric, fibrillary and non-fibrillary Aβ species. Zinc binds to the same site as copper, but binding is again weaker with affinities in the lower micromolar range. The commonly accepted coordination includes Asp1, Glu11 and the three histidines. Zinc immediately precipitates Aβ and NMR structures are deposited only for zinc-mediated dimeric Aβ-zinc (II) species of a mutant human Aβ(1–16) and rat Aβ(1–16) revealing part of this coordination and different dimerization patterns (Figures 4B,C). Aggregation increases the affinity of Aβ for copper and zinc leading to the apparent high concentrations in the amyloids. However, the structural understanding of this process is just at the beginning despite all efforts taken.

In summary, the biometals copper and zinc bind to different folded regions of the APP ectodomain and to various Aβ species. Affinities for all sites are about 100 times higher for copper, which challenges the idea of zinc binding to APP in the synaptic cleft despite its excess. In all complexes the metals seem to be involved in diverse conformational changes of APP and APLP1 as well as APLP2, inducing dimerization or oligomerization in different ways and thus might influence specific physiological and pathological processes.

## The Role of Copper and Zinc Ions in Alzheimer’s Disease

### Zinc and Alzheimer’s Disease

Sustained elevation of zinc levels are detrimental for neurons and have therefore been implicated in AD pathogenesis (Koh et al., 1996). A multitude of studies have analyzed overall zinc levels within AD vs. control brains with rather inconsistent outcome (Ayton et al., 2013). In consensus, however, it has been clearly demonstrated that zinc levels are increased in amyloid plaques of AD patients (Bush et al., 1994c). The elevated zinc levels concern only a small fraction of the total cortical volume, and the total tissue zinc concentrations only rise during advanced pathology (Religa et al., 2006). Closer analysis revealed zinc binding to the residues 6–28 of the Aβ peptide (Bush et al., 1993, 1994a,b,c), where zinc ions may bind to histidines 6, 13 and 14 (Damante et al., 2009) as described above. Binding of zinc ions induces rapid precipitation of Aβ into insoluble aggregates in humans. However, since the rat and mouse Aβ sequences differ from the human Aβ sequence in three amino acids, differences in the affinity for zinc binding may explain why mice and rats do not develop amyloid pathology (Bush et al., 1994c). Zinc is released by glutamatergic fibers in the cortex and hippocampus where the synaptic vesicle located zinc-transporter 3 (ZnT3) loads the metal into synaptic vesicles (Frederickson et al., 2000). In addition to the increased zinc concentrations in amyloid plaques the expression of ZnT3 is closely associated to Aβ deposition. Ten different zinc-transporters have been identified with ZnT3 showing its highest expression within the brain (Palmiter et al., 1996; Huang and Tepaamorndech, 2013). Low ZnT3 expression reduces interstitial (Lee J.-Y. et al., 2002) and vessel-wall (Friedlich et al., 2004) amyloid but increases the levels of soluble Aβ in the brains of the APP transgenic x ZnT3 KO mice (Lee J.-Y. et al., 2002). Zinc does not only affect Aβ aggregation but also influences APP processing and function. APP processing and subsequent Aβ generation is dependent on ADAM10 activity. ADAM10 requires zinc binding for proteolysis and therefore subtle changes in ADAM10 activity influence Aβ production (Lammich et al., 1999).

### Copper and Alzheimer’s Disease

In healthy brain tissue the total copper levels have been reported to increase from youth to adulthood followed by a constant decrease during aging (Maynard et al., 2002). Aged but healthy brain tissue contains approximately 80 μM copper, whereas copper levels are decreased by approx. 30%–40% in affected brain regions of AD patients (Deibel et al., 1996; Adlard and Bush, 2006; Religa et al., 2006; Magaki et al., 2007). In contrast, copper levels (and zinc levels) are enriched in extracellular amyloid plaques (Lovell et al., 1998; Dong et al., 2003; Miller et al., 2006; Leskovjan et al., 2009). Thus, either elevated levels of copper directly associated with the Aβ peptide or decreased levels of copper in amyloid plaques surrounding brain tissue might affect the course of AD. Copper binds with high affinity to Aβ and promotes its oligomerization and neurotoxicity (Atwood et al., 1998, 2004; Huang et al., 1999; Masters and Selkoe, 2012). Moreover, cellular copper deficiency promotes the amyloidogenic processing of APP leading to increased Aβ levels (Cater et al., 2008; Hung et al., 2009), whereas an elevation of intracellular copper levels promotes the non-amyloidogenic pathway and attenuates Aβ generation in cells (Borchardt et al., 1999; White et al., 2006; Donnelly et al., 2008) as well as in transgenic mice (Cherny et al., 2001; Bayer et al., 2003; Phinney et al., 2003; Adlard et al., 2008). However, the molecular mechanisms leading to elevated Aβ production in the presence of decreased cellular copper levels are currently elusive.

It has been suggested that copper induces conformational changes in APP that influence the monomer/dimer equilibrium and thus affects the proteolytic processing of APP (Kong et al., 2008; Baumkötter et al., 2014). In line, it has been shown that enhanced APP dimerization leads to reduced Aβ generation (Kienlen-Campard et al., 2008; Eggert et al., 2009). On the other hand, it has been reported that attenuated APP dimerization correlates with reduced Aβ levels (Munter et al., 2007; Kaden et al., 2008; Richter et al., 2010). Further studies will be required to solve this discrepancy.

An alternative mechanism underlying elevated Aβ production in the presence of decreased cellular copper levels might concern the influence of copper on APP trafficking (Hung et al., 2009; Acevedo et al., 2011), either by altered conformational changes (Spoerri et al., 2012) or more indirectly by regulation of APP phosphorylation (Acevedo et al., 2014). Additionally, copper modifies Aβ and accelerates its aggregation. The copper-induced Aβ oligomerization was found to contain a membrane-penetrating structure (Curtain et al., 2001; Smith et al., 2006). As for other Aβ aggregates, the copper-bound Aβ oligomers complex have been shown to exhibit cytotoxic properties and vice versa general Aβ toxicity in tissue culture is partially dependent on copper (You et al., 2012).

Since more than a decade it is known, that copper may play also a detrimental role in AD due to its interaction with Aβ-peptides leading to amyloid fibrilization, its influence on Tau and GSK3β and induction of oxidative stress (Kenche and Barnham, 2011). Based on these observations Bush and Tanzi (2008) have proposed the “Metal Hypothesis of AD”. Therefore potential therapeutic strategies have been developed either targeting Aβ copper interactions by selectively occupying the metal binding site on Aβ or through development of peptides effectively competing with Aβ-peptides for the metal ions. Such an approach has been developed e.g. for Wilson’s disease (WD) to target a metal overload using metal chelators. Such chelators as desferrioxime, penicillamine and trientine have very high metal binding affinities and are hydrophilic. Therefore such chelators are inappropriate to tackle a brain disease like AD since they will not be able to cross the blood brain barrier (BBB). In contrast to these hydrophilic compounds metal protein attenuating compounds have been developed like clioquinol (CQ, 5-chloro-7-iodo-8-hydroxyquinoline), which are indeed capable to cross the BBB. Although some approaches have been promising in early clinical trials none of these compounds have clinically proven to be effective in AD (Adlard et al., 2008; Lannfelt et al., 2008). One potential drawback might be the unwanted side effects on APP copper interactions and its subsequent physiological consequences.

## Copper and Zinc in the Interplay with APP at the Synapse

Based on the observation that zinc and copper bind APP, APLP1 and APLP2 with high affinities and the high abundance of these metals in the synaptic cleft during neurotransmission, the impact of copper and zinc on the trans-cellular dimerization properties of APP during cell adhesion and synaptogenesis went into focus of current research.

### Influence of Copper on Brain Function

The transition state metals copper and zinc, as well as iron, are essential for the catalytic activity of a variety of enzymes, including amongst others the cuproenzymes cytochrome c oxidase, superoxide dismutase (SOD), ceruloplasmin, tyrosinase and dopamine β hydroxylase (Turski and Thiele, 2009). However, copper is also toxic to cells and thus, its distribution in the cell has to be tightly regulated. In neurons, the copper transporter 1 (CTR1) mediates copper import (Lutsenko et al., 2010). Interestingly, only reduced copper (I) is transported by CTR1 (Macreadie, 2008). Thus, extracellular copper (II) has to be reduced to copper (I) by membrane bound metalloreductases prior to uptake into the cell by CTR1. The assumed mammalian metalloreductase remains to be identified (Lee J. et al., 2002). Potential candidates are the Steap proteins (Ohgami et al., 2006) as well as APP since it binds with high affinity to copper and is also able to reduce copper (Multhaup et al., 1996). However, elevated APP levels cause a decrease and APP depletion an increase of intracellular copper concentrations (White et al., 1999; Maynard et al., 2002, 2006; Phinney et al., 2003; Bellingham et al., 2004; Treiber et al., 2004), arguing against a function of APP in copper influx.

Copper imported via CTR1 is delivered by specific copper chaperones directly to different cuproenzymes. For example, the copper chaperone CCS mediates transfer of copper from CTR1 to the SOD (McCord and Fridovich, 1969; Culotta et al., 1997). Delivery of copper to cuproenzymes in the secretory pathway is mediated by the copper chaperone ATOX1, which transfers copper (I) from CTR1 to the intracellular copper transporters ATP7A and ATP7B, located in different intracellular compartments (Kim et al., 2008). Besides these principal metallochaperone mediated copper transport pathways, cytoplasmic copper also binds to glutathione immediately after entry into the cell and is subsequently transferred to metallothionein (Hung et al., 2010). Due to the described mechanisms, the intracellular concentration of free copper is maintained at exceedingly low levels (Rae et al., 1999).

Copper is found all over the brain and is most abundant in the basal ganglia (Madsen and Gitlin, 2007). The data on chelatable copper concentrations in extracellular fluids and intracellular compartments is a matter of debate. In some particular neurons copper is released at the synapse (Hartter and Barnea, 1988; Brown et al., 1997), estimated to reach upon depolarization and activation of N-methyl-D-aspartate (NMDA) receptors (Rajan et al., 1976; Hartter and Barnea, 1988; Kardos et al., 1989; Peters et al., 2011; Tamano and Takeda, 2011) micromolar concentrations (Kardos et al., 1989) to approx. 15 μM (Hopt et al., 2003). Moreover, transient copper concentrations above 100 μM at the synaptic cleft have been reported (Kardos et al., 1989; Gaier et al., 2013). Consistently, the synaptic cleft is the only reported microenvironment within the brain where chelatable copper might be easily excessible (Roberts et al., 2012). Interestingly, the activation of NMDA receptors leads to relocalization of ATP7A from the trans golgi-network (TGN) to neuronal processes and thus in turn contributes to increased copper concentrations at the synapse (Schlief et al., 2005).

Loss-of-function mutations in copper transporters, such as ATP7A and ATP7B, lead to hereditary diseases, Menkes disease (MD), or WD. Loss of ATP7A function causes growth failure, brittle hair, hypopigmentation, arterial tortuosity and neuronal loss most prominent in the hippocampus and cerebellum (Okeda et al., 1991; Chelly et al., 1993; Mercer et al., 1993; Vulpe et al., 1993). The phenotypes are mostly due to consequences of specific cuproenzymes dysfunction resulting from reduced cellular copper levels (D’Ambrosi and Rossi, 2015). Loss of ATP7B, which is primarily expressed in the liver, leads to copper overload in the liver and later in the brain, possibly due to impaired copper transport at the BBB (Huster and Lutsenko, 2007; Kaler, 2011). Although copper levels are mainly increased in cerebrospinal fluid and in the basal ganglia (Südmeyer et al., 2006), WD patients show widespread neuronal cell loss and white matter abnormalities, causing symptoms that include parkinsonism, seizures and mental disorders (Gitlin, 2003).

The molecular mechanisms underlying copper induced neurodegeneration are only partially understood. Mechanism(s) discussed involve S-nitrosylation, oxidation and allosteric modulation, increased anchorage of the neurotransmitter receptors to the membrane, and modulation of neurotransmitter receptor function (Weiser and Wienrich, 1996; Kim and Macdonald, 2003; Schlief and Gitlin, 2006; El Meskini et al., 2007; Huidobro-Toro et al., 2008; Peters et al., 2011; Gaier et al., 2013).

### Influence of Zinc on Brain Function

In contrast to copper ions there is a substantial amount of zinc loosely bound to biomolecules, designated as reactive or chelatable zinc, which is implicated in neuronal signaling. Reactive zinc is largely distributed within presynaptic vesicles in some axon terminals throughout the telencephalon and co-localizes with a subset of glutamatergic neurons (Frederickson et al., 2000). Cytosolic reactive zinc levels are in the picomolar range and are estimated to rise to micromolar levels in the synaptic cleft and in intracellular compartments, such as mitochondria, secretory vesicles and lysosomes (Sensi et al., 2009). While it is evident that zinc is released during synaptic activity, there is little consensus on the amount or duration of its existence in the synaptic cleft (Watt et al., 2010). Zinc homeostasis is mainly maintained by regulated activities of “zinc importing” ZnT transporters (SLC30 family), “zinc exporting” ZIP transporters (SLC39 family), and zinc buffering proteins, including metallothioneins (Sensi et al., 2011). At the synapse, vesicular released zinc interacts with various neuronal ion channels (NMDA, (α-amino-3-hydroxy-5-methyl-4-isoxazolepropionic acid, AMPA), GABA_A_ (γ-aminobutyric acid type A) receptors), Glycine and other surface receptors, such as TrkB. Furthermore, zinc can bind to and regulate ProSAPs/Shanks scaffolding proteins of the postsynaptic density (PSD), involved in synaptic signaling. Indeed, in some cerebral areas nearly 50% of the glutamatergic synapses are actually “glu-zinc-ergic” (Watt et al., 2010). Therefore, zinc is considered as an important synaptic modulator, affecting neurotransmission at inhibitory as well as excitatory synapses.

### Influence of Copper/Zinc on APP Synaptic Function

Synaptic function of APP is discussed in detail in other chapters of this special issue. Briefly, loss of APP function leads to a reduced number of dendritic spines (Watt et al., 2010; Tyan et al., 2012) and to impairments in the structural plasticity (Zou et al., 2016). A possible mechanism infers an important trans-synaptic adhesion molecules like function for membrane anchored APP (Siddiqui and Craig, 2011) and major neurotrophic roles of the secreted sAPPα ectodomain (Soba et al., 2005; Bell et al., 2008; Jimenez et al., 2011; Aydin et al., 2012; Caldwell et al., 2013; Baumkötter et al., 2014). Loss of APLP2 (von Koch et al., 1997) had no consequences on brain function (Weyer et al., 2011, 2014; Midthune et al., 2012), whereas APP/APLP2 and APLP1/APLP2 double KO mice exhibit severe deficits in formation of the neuromuscular junction and die early after birth. This suggests that APP/APLPs share some overlapping functions, but also have distinct synaptic functions that are not compensated by the other family members.

**Figure 5:**
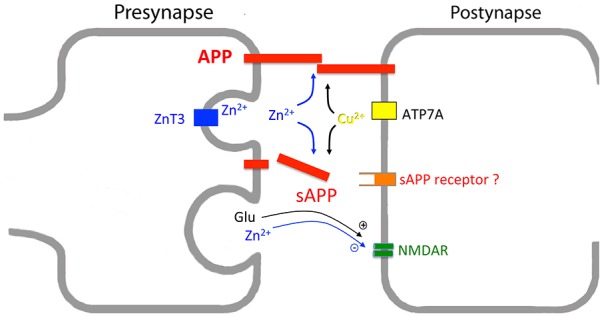
**Interplay of synaptic copper, zinc and APP/APLPs.** Scheme showing postsynaptic copper and presynaptic zinc release by ATP7A and zinc-transporter 3 (ZnT3), respectively. APP/APLPs (here indicated as APP) either form trans-synaptic dimers or are secreted into the synaptic cleft. APP processing, APP trans-dimerization, and possibly also binding of sAPP to a putative receptor are modulated by copper and zinc levels. As release of these metal ions depends on synaptic activity, structural changes within APP/APLPs might be accompanied with copper and zinc binding. Concomitantly, metal binding seems to function as a sensor to strengthen or destabilize APP oligomers. At glutamatergic synapses a key player in this process is the N-methyl-D-aspartate (NMDA) receptor, with both sAPP and zinc affecting NMDAR function. In addition, the loss of APP goes along with a decrease in D-Serine, which acts as a co-agonist on NMDAR. The mechanism underlying altered D-Serine secretion has not yet been determined.

As pointed out before, copper binds APP at different sites within the E1 and E2 domain, causing structural changes and altered dimerization properties and heparin binding characteristics (Baumkötter et al., 2012). Copper binding to the GFLD coincides with structural rearrangements in the heparin-binding loop region (Baumkötter et al., 2014), possibly also representing an APP dimerization interface (Kaden et al., 2008). In line, copper-induced trans-directed *in vitro* interaction of APP, and mutations, abolishing copper-binding to the GFLD, reduce APP synaptogenic activity in a cellular assay system (Baumkötter et al., 2014). Therefore, it appears reasonable that copper might also modulate the neurotrophic function of secreted APP forms. Thus, copper modulation in the synaptic cleft upon synaptic activity might contribute to APP trans-synaptic signaling in context of synaptic maturation (Figure 5). Actually, a decrease of D-serine in brains of APP knockout mice was reported to contribute to synaptic deficits in aged mice (Zou et al., 2016). Most likely the decrease in D-serine is explained by a loss of function of APP in calcium-dependent release of D-serine from astrocytes (Martineau et al., 2014). Thus, as both D-serine and zinc can bind and modulate NMDA receptor function in antagonistic ways, it is tempting to speculate that APP might sense changes in zinc concentrations that in turn could affect D-serine secretion in the synaptic cleft, antagonizing the antidepressant-like effects of zinc and thereby contributes to homeostasis of synaptic activity. A validation of this tempting hypothesis is however hampered by the technical limitation for the quantification of reactive copper in the synaptic cleft. Knock-in approaches testing different APP mutants might be a way to disentangle the functional relation between copper and APP at the synapse.

Different reports suggest binding of APP, APLP1 and to a minor extent also APLP2 to zinc with affinities in the low micromolar range (Bush et al., 1993; Mayer et al., 2014, 2016) and it was shown by FRET analysis of heterologous expressed APP/APLPs that addition of zinc can induce clustering of APP, APLP1 (EC50: 10 μM), and APLP2 (EC50: 300 μM) as well as of all types of heterotypic APP/APLPs combinations (Mayer et al., 2016). Interestingly, combinations of APLP1 with APLP2 also exhibited an EC50 at about 50 μM. As reactive zinc concentrations can reach high micromolar and possibly also millimolar concentrations in the synaptic cleft, all types of oligomers (including homo- and heterotypic APLP2 containing oligomers) are likely formed also under *in vivo* conditions at the synapse. Consistently, addition of 50 μM zinc to cells expressing APLP1 caused a lateral concentration at cell-cell contact sites (Mayer et al., 2016), as formerly described by Soba et al. (2005) in presence of copper (Baumkötter et al., 2014). Notably, alanine mutations of one of four histidines involved in zinc binding at the M3 site (H430/H433/H450/H452) did not abolish zinc-induced oligomerization and only lowered the affinity about two fold. This suggests that APP and APLP2 trans-directed dimerization might also be affected by zinc in a similar way, possibly in interplay with heparin binding and APP/APLPs dimerization/oligomerization (Bush et al., 1994a).

Despite some major gaps in our understanding of APP/APLPs synaptic function, the current data as presented in this review article strongly suggest that activity-dependent changes in zinc and copper concentrations in the synaptic cleft can be sensed by the APP/APLP family. In turn, they seem to modulate neurotransmission by different pathways including neurotrophic activity of sAPPα or trans-cellular dimerization/signaling. However, one major gap in our current understanding, especially in respect to the function of copper, is the limitation of available sensitive sensors, allowing determination of local transient changes in copper concentration. In this regard, live-cell optical imaging with fluorescent sensors offers a potentially powerful approach for interrogating aspects of labile copper accumulation, speciation, trafficking, and redox function in living systems at the molecular level. Such reagents have greatly facilitated the study of calcium and zinc in cell biology, but analogs tools for cellular copper remain underdeveloped (Zeng et al., 2006; Dean et al., 2012). Therefore, the most promising way might actually be, to use mutant APP impaired in copper and/or zinc binding in different cellular assays, allowing to estimate the pathophysiological impact of copper and zinc on APP function and its role in AD.

## Author Contributions

KW, AA, CUP and SK wrote this review article.

## Conflict of Interest Statement

The authors declare that the research was conducted in the absence of any commercial or financial relationships that could be construed as a potential conflict of interest.
